# A Year at the Forefront of Engineering Photosynthesis

**DOI:** 10.1242/bio.059335

**Published:** 2022-07-25

**Authors:** Sophie L. Johnson

**Affiliations:** Department of Plant Sciences, University of Oxford, Oxford, OX1 3RB, UK

**Keywords:** Engineering photosynthesis, Synthetic biology

## Abstract

Multiple proof-of-principle experiments and successful field trials have demonstrated that engineering photosynthesis is a viable strategy for improving crop yields. Advances to engineering technologies have accelerated efforts to improve photosynthesis, generating a large volume of published literature: this Review therefore aims to highlight the most promising results from the period February 2021 to January 2022. Recent research has demonstrated the importance of understanding the impact of changing climates on photosynthesis to ensure that proposed engineering strategies are resilient to climate change. Encouragingly, there have been several reports of strategies that have benefits at temperatures higher than current ambient conditions. There has also been success in engineering synthetic bypass pathways, providing support for the feasibility of a synthetic biology approach. Continued developments in all areas of engineering photosynthesis will be necessary for sustainably securing sufficient crop yields for the future.

This article has an associated First Person interview with the first author of the paper.

## Abstract (precis)

**Summary:** An a Year at the Forefront Review highlighting promising advances in engineering photosynthesis made in the period February 2021 to January 2022.

## Introduction

The growing global population and increasing demand for bioenergy are predicted to result in future crop yields falling substantially behind demand without significant improvements to productivity (Ray et al., 2013). Future yield shortages will be further exacerbated by increasing temperatures resulting from climate change, with large losses for major crops such as maize, rice and soybean predicted (Jägermeyr et al., 2021). International efforts are therefore underway to improve crop yields, notably by targeting photosynthetic efficiency (Ort et al., 2015; Kubis and Bar-Even, 2019; Batista-Silva et al., 2020). Indeed, theoretical yield potential calculations have shown that improving photosynthesis is the only viable option to achieve the necessary yield improvements given the plateauing benefits of the Green Revolution (Murchie et al., 2009; Zhu et al., 2010; Long et al., 2015). Multiple strategies have been explored to this end, including targeting light capture efficiency, optimising photosynthetic enzymes, introducing a carbon-concentrating mechanism (CCM) or alternative form of photosynthesis to C3 species, optimising photorespiration, and engineering a smart canopy, with the aim of attaining sustainable crop improvements.

Targeting light capture is a major strategy for engineering photosynthesis. The light reactions of photosynthesis convert light to chemical energy through the fixation of atmospheric carbon dioxide: subsequent carboxylation of ribulose 1,5-bisphosphate (RuBP) by the enzyme ribulose 1,5-bisphosphate carboxylase/oxygenase (Rubisco) results in the generation of 3-phosphoglycerate (3PGA), which can be integrated into the Calvin–Benson–Bassham cycle and converted to organic sugars. Light capture depends on light-harvesting complexes arranged around reaction centres that form photosystems for the absorption of specific wavelengths. Light harvesting therefore makes partial use of the available solar spectrum. Moreover, only a fraction of the theoretical maximum of 12% solar energy conversion efficiency is achieved since plants are subjected to fluctuating light conditions and tend to absorb more light than can be productively used (Blankenship et al., 2011). In addition, although photoprotective mechanisms prevent damage to the photosystems at saturating light levels, slow downregulation of these processes can limit photosynthetic efficiency (Kromdijk et al., 2016). These limitations to photosynthesis could be overcome by (i) engineering pigments/light-harvesting complexes to access a wider range of the solar spectrum, for example as proposed by the PhotoRedesign consortium (Hitchcock et al., 2021), and (ii) improving the efficiency of protective mechanisms.

In addition, an alternative strategy is to target Rubisco itself. Rubisco is an inefficient enzyme with a low catalytic rate and low CO_2 ­_affinity (Flamholz et al., 2019) that is poor at distinguishing CO_2_ from O_2_, particularly at elevated temperatures (Sage and Kubien, 2007). This promiscuity results in a competing oxygenation reaction at the Rubisco active site, generating toxic 2-phosphoglycolate (2PG) that must be recycled via the energetically costly photorespiratory pathway (Zhu et al., 2010; Walker et al., 2016; Busch, 2020). Further strategies to improve photosynthesis therefore include engineering of Rubisco to increase its CO_2_ specificity, introducing a CCM, or engineering a novel synthetic photorespiratory bypass. For example, transgenic tobacco plants expressing the *E. coli* glycolate oxidation pathway had increased photosynthesis and biomass compared with wild type (South et al., 2019). Novel synthetic biology approaches have also been proposed (Bar-Even et al., 2010; Schwander et al., 2016; Miller et al., 2020), and their implementation is rapidly becoming feasible with improving technologies (e.g. Scheffen et al., 2021). This Review aims to highlight the most promising advances in engineering photosynthesis in the period February 2021 to January 2022 (Table 1).

**Table 1.**
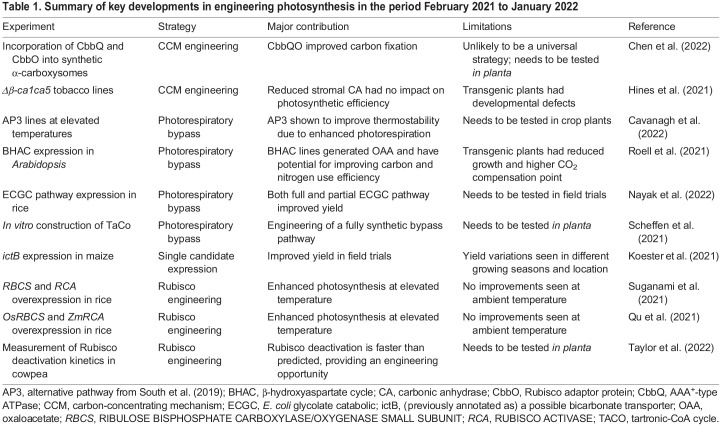
Summary of key developments in engineering photosynthesis in the period February 2021 to January 2022

## A year at the forefront of engineering photosynthesis

### Discoveries

Engineering of photosynthesis relies on the development of molecular and synthetic biology approaches that are guided by detailed molecular knowledge. Advances in understanding the fundamental processes in different organisms therefore represent a significant contribution towards engineering photosynthesis. Progress has been made in both fundamental mechanistic understandings and technologies to implement engineering strategies in the period February 2021 to January 2022.

#### Cyanobacterial CCM engineering

**Fig. 1.**
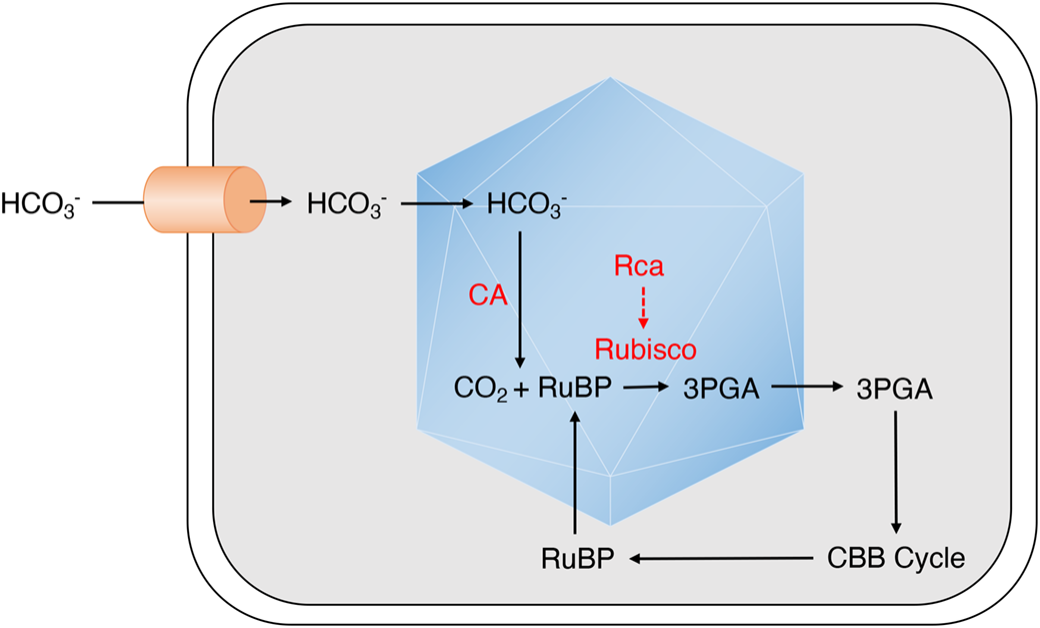
**Simplified cyanobacterial CMM.** Hydrogen carbonate (HCO_3_^−^) transporters (orange) in the membrane of cyanobacteria import HCO_3_^−^ that is concentrated in proteinaceous carboxysomes (blue). Rubisco catalyses the carboxylation of ribulose 1,5-bisphosphate, generating 3-phosphoglycerate, a Calvin–Benson–Bassham (CBB) cycle substrate. Some species require Rubisco activase to activate Rubisco (red dashed arrow). 3PGA, 3-phosphoglycerate; CA, carbonic anhydrase; Rca, Rubisco activase; Rubisco, ribulose 1,5-bisphosphate carboxylase/oxygenase; RuBP, ribulose 1,5-bisphosphate.

C_3_ yields are predicted to be enhanced by up to 60% by introduction of a cyanobacterial CCM into chloroplasts (McGrath and Long, 2014; Yin and Struik, 2017). All cyanobacterial CO_2_ fixation is enhanced by protein complexes called carboxysomes that consist of Rubisco, carbonic anhydrase (CA), a nucleating protein, and often Rubisco activase (Rca) (reviewed in Turmo et al., 2017; Hennacy and Jonikas, 2020). Inorganic HCO_3_^−^ transporters facilitate CO_2_ concentration within the cell, resulting in elevated CO_2_ concentrations in the carboxysomes and improved Rubisco carbon fixation (Fig. 1) (Price, 2011). Despite the recent reconstitution of a minimal carboxysome in flowering plants (Long et al., 2018), a functional plant CCM is yet to be demonstrated, suggesting that additional molecular components are required. It is possible that Rubisco requires chaperones for folding and activation in this context (Long et al., 2018). Indeed, a new discovery is that Rca components CbbQ and CbbO can increase overall carbon fixation when engineered into the α-carboxysomes of *Halothiobacillus neapolitanus* (Chen et al., 2022), although the dependence of carboxysome-localised Rubisco on activase activity is hypothesised to vary between organisms (Tsai et al., 2022). In addition to optimising Rubisco activity, functionalisation of an engineered chloroplast CCM will also require the elimination of native stromal CA to allow for the accumulation of HCO_3_^−^ within carboxysomes (Dean Price et al., 2011). Hines et al. recently demonstrated the feasibility of this by knocking out the major stromal isoforms β-CA1 and β-CA5 in tobacco. Mutant plants exhibited no photosynthetic defects, instead showing developmental perturbations that included low germination rates, accumulation of necrotic lesions and early cessation of flowering (Hines et al., 2021). The authors hypothesised that this was the result of disrupted bicarbonate biosynthesis, the effects of which would be mitigated by the exogenous expression of bicarbonate transporter(s) in engineered CCMs (Hines et al., 2021). Generation of a fully functional C_3_ CCM could therefore be a realistic prospect.

#### Synthetic biology approaches to optimising photorespiration

**Fig. 2.**
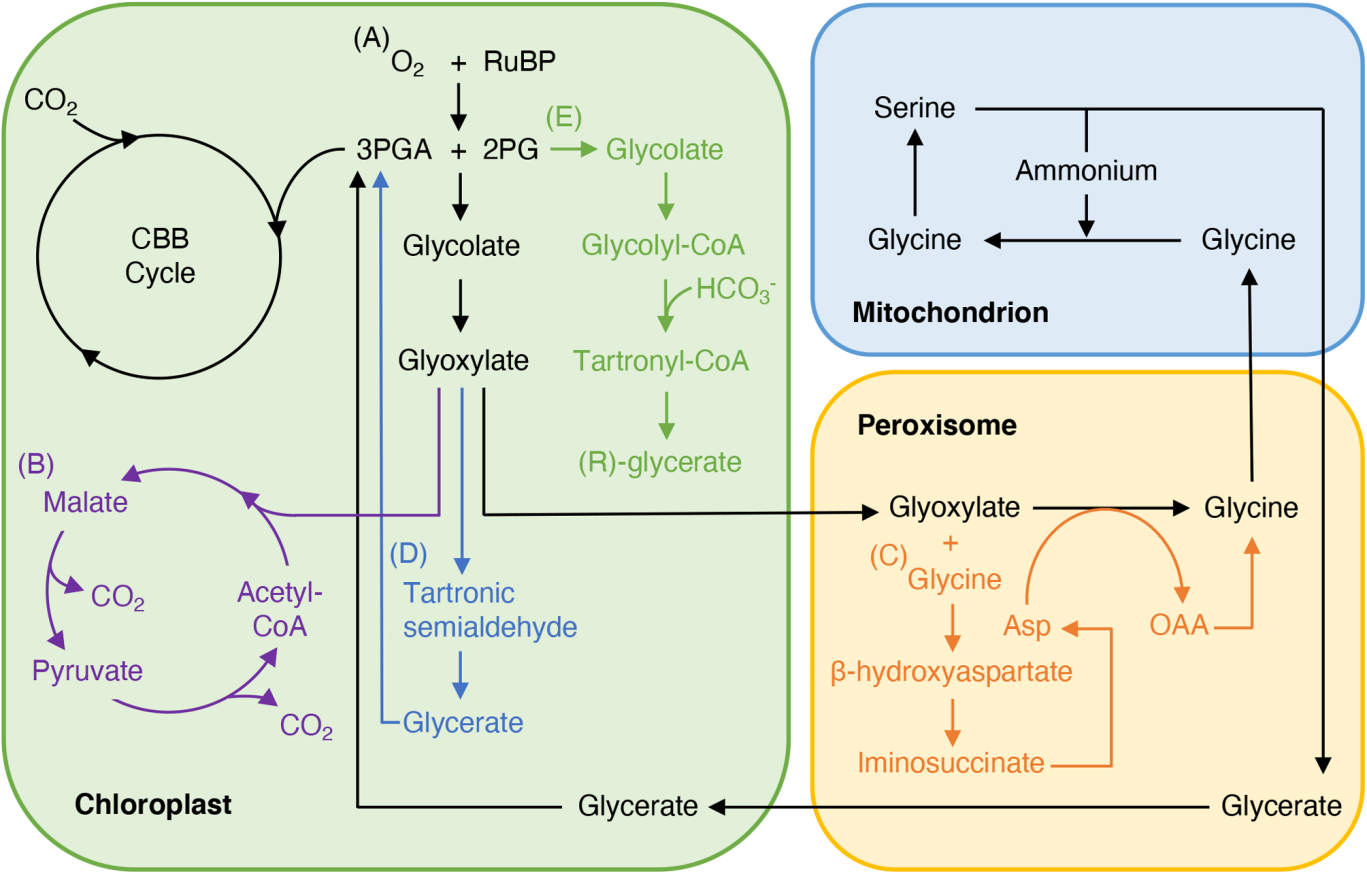
**Integration of the Calvin–Benson–Bassham cycle with native photorespiration and different photorespiratory bypasses.** (A) Rubisco oxygenation generates 2-phosphoglycolate that is recycled via the native photorespiratory pathway (black). (B) Alternative pathway 3 (purple) (South et al., 2019; Cavanagh et al., 2021) bypasses photorespiration by metabolising glycolate in the chloroplast. (C) The β-hydroxyaspartate cycle (orange) (Roell et al., 2021) generates oxaloacetate from glycolate while conserving more carbon and nitrogen than native photorespiration. (D) *E. coli* glycolate catabolic pathway (blue) (Nayak et al., 2022) generates 3-phosphoglycerate for Calvin–Benson–Bassham cycle metabolism without requiring catalytic steps in additional compartments to the chloroplast. (E) Fully synthetic tartronyl-CoA pathway (green) (Scheffen et al., 2021) provides a more direct route for glycolate assimilation than native photorespiration. Enzymes, stoichiometries and co-substrates have been omitted for clarity. 2PG, 2-phosphoglycolate; 3PGA, 3-phosphoglycerate; Asp, aspartate; CBB, Calvin–Benson–Bassham; OAA, oxaloacetate; RuBP, ribulose 1,5-bisphosphate.

In the absence of a CCM, photosynthetic efficiency is limited by photorespiration (Fig. 2A), which has the potential to be improved for energy and carbon use efficiencies (Shen et al., 2019; South et al., 2019). Building on the success of the introduction of a synthetic alternative pathway (AP3) in tobacco to increase biomass (Fig. 2B) (South et al., 2019), Cavanagh et al. tested whether AP3 could confer improved thermal tolerance. Notably, higher photosynthetic rates were found to be maintained in AP3 plants under short-term temperature stress, resulting in transgenic plants retaining 19% more biomass than wild-type plants under the same conditions (Cavanagh et al., 2022). No increase in maximum carboxylation or electron transport rate was observed in the transgenic lines, consistent with the observed thermostability being conferred by improved photorespiration (Cavanagh et al., 2022). Alternative photorespiratory bypass strategies based on the introduction of the β-hydroxyaspartate cycle (BHAC) into *Arabidopsis thaliana* or the *Escherichia coli* glycolate catabolic (ECGC) pathway into rice were met with more variable success (Roell et al., 2021; Nayak et al., 2022) (Fig. 2C,D). The BHAC from marine proteobacteria oxidises glycolate to glyoxylate, which is then converted to oxaloacetate (OAA) via four enzymatic steps without the loss of carbon or nitrogen (in contrast to native photorespiration). Four BHAC enzymes were introduced into *Arabidopsis*, targeted to the peroxisome, and shown to function *in planta* to the extent that relevant metabolic intermediates could be detected. However, transgenic plants had reduced growth compared to wild type and BHAC was not found to improve the CO_2_ compensation point (Roell et al., 2021). Despite this, BHAC lines have a potential use in engineering C_4_ photosynthesis (an alternative CCM) as they accumulate key C_4_ metabolite OAA without requiring the establishment of phosphoenolpyruvate-dependent CO_2_ fixation (Roell et al., 2021). Introduction of the ECGC pathway into rice involved constitutive expression of five genes in rice chloroplasts (Nayak et al., 2022). Transgenic plants expressing both the full (FB) and partial (PB) ECGC pathway were found to maintain higher CO_2_ assimilation rates and growth than wild type, which translated into up to 46.7% and 67.0% yield increases in FB and PB plants, respectively (Nayak et al., 2022). Introduction of orthogonal metabolic pathways to bypass photorespiration therefore has real potential for improving crop yields.

A significant advance in the period under review has been the use of state-of-the-art enzyme engineering and directed evolution methods to make the previously hypothetical tartronyl-CoA (TaCo) photorespiratory bypass a reality (Fig. 2E). Theoretical calculations suggested that interfacing TaCo with photorespiration would increase the carbon efficiency of the Calvin–Benson–Bassham cycle from 75 to 100%, while using 21% less ATP and 29% less reducing equivalents than native photorespiration (Scheffen et al., 2021). Tartronyl-CoA, formed by the carboxylation of glycolyl-CoA, and the reactions of the TaCo pathway are not known to occur in nature, so Scheffen et al. identified *Methylorubrum extorquens* propionyl-CoA carboxylase *Me*PCC as a potential candidate for engineering TaCo. Structure-guided rational design was used to engineer the substrate preference of *Me*PCC, resulting in a glycolyl-CoA carboxylase (GCC) that had a 15-fold increase in catalytic efficiency towards glycolyl-CoA. Directed evolution using a high-throughput approach based on a novel microfluidics screen identified a GCC variant with catalytic efficiency of 3.6×10^4^ M^−1^ s^−1^ for glycolyl-CoA carboxylation and reduced futile ATP hydrolysis. Tests *in vitro* found that reconstituted TaCo could generate the physiologically relevant glycerate stereoisomer, suggesting that TaCo has the potential to be successfully implemented *in planta* (Scheffen et al., 2021). Realisation of TaCo in plants remains to be achieved, but the results so far have provided a clear proof-of-principle for the engineering of fully synthetic pathways to improve photosynthesis.

### Technological innovations and new resources

Efforts to engineer photosynthesis are limited by available technologies, particularly for stable nuclear transformation of large, multigene constructs (>10 genes) into nuclear genomes (Ort et al., 2015) and for the transformation of chloroplast genomes (Bock, 2015; Jackson et al., 2021). However, introduction of point mutations to plastome-localised *RbcL* via a restriction enzyme-based system has been re-demonstrated with a 40% editing efficiency (Lin et al., 2021). Improvements have also been made to an already established proteoliposome system for testing of photoprotective strategies (Nicol and Croce, 2021).

Development of open access, online resources is crucial for photosynthesis research and related engineering strategies (Zhu et al., 2020). This is particularly important for comparing results between labs. For example, Rubisco kinetics were found to vary significantly depending on the lab and method, with k_cat_ and K_c_ found to vary >200% in vascular plants (Iñiguez et al., 2021). A normalised Rubisco kinetic database and methods for correcting empirical values have therefore been proposed and will be invaluable for future Rubisco engineering (Iñiguez et al., 2021). Recently published genome and transcriptome data for species with high photosynthetic efficiencies will also help to inform future research (Xi et al., 2021; Garassino et al., 2022 preprint), as will an analysis of photosynthetic and morphological traits in widely used *indica* rice (Acevedo-Siaca et al., 2021).

Implementation of future design strategies is also likely to require novel promoters with well-defined characteristics (strength, tissue specificity, etc.). A comprehensive analysis of all core promoters in *Arabidopsis*, maize and sorghum revealed determinants of promoter strength (Jores et al., 2021). This allowed for the design of synthetic promoters with chosen characteristics and informed the generation of a computational model to predict promoter strength (Jores et al., 2021). This is likely to make an impact in the future of photosynthetic engineering projects, as well as in other biological engineering contexts.

### New hypotheses

The results produced in the period February 2021 to January 2022 have informed or challenged previous hypotheses. For example, Hines et al. demonstrated that loss of stromal CA activity does not result in photosynthetic defects, contrary to the hypothesis that removal of stromal CA would significantly reduce mesophyll conductance and consequently photosynthesis (Tholen and Zhu, 2011). There has also historically been confusion as to the role of IctB in cyanobacterial CCMs. IctB was originally identified as a bicarbonate transporter (Bonfil et al., 1998) but was subsequently shown not to contribute to bicarbonate transporter loss-of-function lines (Shibata et al., 2002; Xu et al., 2008). If IctB is not a bicarbonate transporter, previous suggestions that *ictB* expression in C_3_ species enhances photosynthesis by facilitating a CCM (Lieman-Hurwitz et al., 2003; Simkin et al., 2015; Gong et al., 2015; Hay et al., 2017) must be overlooking an alternative function. *ictB* expression in a C_4_ species should therefore recapitulate the enhanced photosynthesis phenotype of *ictB* expression in C_3_ plants (Koester et al., 2021). In agreement with this, transgenic *RbcS::ictB* maize lines had a 3.49% increase in yield compared to controls in field trials, with the increased performance attributed to enhanced carbohydrate production (Koester et al., 2021). Yield increase was associated with an increase in photosystem II operating efficiency, suggesting that enhanced photosynthesis was responsible for the improvements (Koester et al., 2021). These results support the hypothesis that IctB is not a CCM bicarbonate transporter: a complete understanding of how CCMs operate therefore remains lacking.

It has long been hypothesised that overexpression of Rubisco could enhance photosynthesis; however, Rubisco overexpression has been associated with a decrease in Rubisco activation and lower Rca:Rubisco ratio (Suzuki et al., 2009; Suganami et al., 2018). Two groups therefore recently tested the effect of Rubisco and Rca co-overexpression. Although the rate of CO_2_ assimilation in Rubisco/Rca-overexpressing rice lines (*RBCS-RCA-ox*) was similar to wild type under ambient conditions, CO_2_ assimilation was enhanced in *RBCS-RCA-ox* lines at 32–36°C (Suganami et al., 2021). Similar results were found with the co-overexpression of *OsRBCS* and *ZmRCA* in rice, with a 26% increase in dry biomass found in transgenic plants compared with wild type when grown at 40°C (Qu et al., 2021). In addition, Rubisco reactivation on shade–sun transitions is known to be slow (Sassenrath-Cole et al., 1994; Tanaka et al., 2019), providing an opportunity for engineering. Notably, a recent paper that determined *in vitro* and *in vivo* Rubisco deactivation half-times in cowpea found faster Rubisco deactivation than previously predicted from wheat values (Taylor et al., 2022). Furthermore, the speed of Rubisco response to sun–shade transitions differed more than Rubisco induction on shade–sun transitions, leading the authors to hypothesise that Rubisco deactivation could be a new target for engineering (Taylor et al., 2022). The significance of this finding will likely be tested in transgenic plants in the future.

### Future prospects

Despite the bleak outlook on future crop security, engineering photosynthesis is a promising approach for improvement, particularly in the light of the recent success of synthetic biology approaches. Given that photosynthesis is a complex trait, it is challenging to predict which strategy will yield the best result: it therefore seems astute to continue developing all avenues of photosynthetic research to ensure a robust strategy for sustainably safeguarding yields in a changing climate. Indeed, engineering strategies are not mutually exclusive, and a combined approach may prove to be the most effective.
